# How Does a Group Reflection Intervention (Schwartz Rounds) Work within Healthcare Undergraduate Settings? A Realist Review

**DOI:** 10.5334/pme.930

**Published:** 2023-12-19

**Authors:** Duncan Hamilton, Cath Taylor, Jill Maben

**Affiliations:** 1 School of Health Sciences, Faculty of Health and Medical Sciences, University of Surrey, UK

## Abstract

**Introduction:**

Schwartz Rounds (“Rounds”) are a confidential group reflection forum, increasingly adopted to support pre-registration healthcare students. This realist review aims to understand what the available literature and key informant interviews can tell us about Rounds in this setting, asking what works, for whom, in what circumstances, and why?

**Methods:**

Published literature discussing Rounds in undergraduate settings were analysed using realist methods to describe how, for whom and in which contexts Rounds work. Four key informants were interviewed using realist methods, to further develop, test and refine a programme theory of Rounds in undergraduate settings.

**Results:**

We identified five core features and five contextual adaptations.

Core: Rounds provide a reflective space to discuss emotional challenges; Rounds promote an open and humanised professional culture; Rounds offer role-modelling of vulnerability, enabling interpersonal connectedness; Rounds are impactful when focused on emotional and relational elements; Rounds offer reflective insights from a wide range of perspectives.

Contextual adaptations: Rounds allow reflection to be more engaging for students when they are non-mandatory; perceptions of safety within a Round varies based on multiple factors; adapting timing and themes to students’ changing needs may improve engagement; resonance with stories is affected by clinical experience levels; online adaptation can increase reach but may risk psychological safety.

**Discussion:**

Schwartz Rounds are a unique intervention that can support healthcare students through their pre-registration education. The five “core” and five “contextual adaptation” features presented identify important considerations for organisations implementing Rounds for their undergraduates.

## Introduction

Healthcare students face a wide array of challenges throughout their undergraduate training, including maintaining their personal and professional values [1], managing their mental health and wellbeing [2,3], developing interpersonal skills [4,5], and constructing a professional self-identity that can survive and thrive within the culture of healthcare organisations [6,7]. Although much the same could be said for students studying any course, healthcare students spend a significant amount of their time in clinical practice (e.g. 800 hours for student nurses in Australia [8], up to 2,300 hours in much of Europe [9]), exposed to the complex (and sometimes traumatic) nature of healthcare practice, while being expected to perform to high academic and clinical standards.

Consequently, values-based recruitment in the United Kingdom (UK) [10] suggests that undergraduates need to be selected for intrinsic caring values. However, values such as empathy may be at risk of eroding over time [11,12]. Students’ values may be challenged, compromised or even crushed, on exposure to the complex realities of clinical practice [1,13,14].

Clinical educators recognise that students can possess high levels of perfectionism and that they have a duty of care with regard to students’ risks of anxiety, stress, depression and burnout [15], with nursing students often seeking informal sources of counselling to cope with placement stress, such as peers, family and friends [16]. Supporting the wellbeing of students is considered to be an important component of responding to course attrition rates, which may be 25% or higher for disciplines such as adult nursing [17] and now more important than ever for a post-pandemic context.

Schwartz Rounds (“Rounds”) are a group reflection intervention that has a proven evidence base in addressing some of these concerns in healthcare staff in professional care settings [18,19]. In a (typically) monthly group forum, staff (clinical and non-clinical) openly discuss emotional, social and ethical challenges of professional care-giving in a confidential, facilitated environment.

Rounds originated in the United States, where a patient, Kenneth Schwartz, wrote an article in the Boston Globe describing how “small acts of kindness made the unbearable bearable” in the care he received during a terminal illness [20], but that some staff weren’t always able to enact this kindness. This led Schwartz to reflect on what it was like to work in such challenging care environments. Following his death, the Schwartz Center for Compassionate Care was established. This is where Rounds were first developed, encompassing the insights from Schwartz during his illness and the healthcare experiences of his oncologist and oncology nurse, on the value of compassion and connection, and how this might be supported in staff [21].

Rounds were brought to the UK in 2009 by the Point of Care Foundation (POCF) (at that time part of the King’s Fund) and have proliferated in the UK [19], expanding to settings such as hospices [22] and medical schools [23].

In a UK national evaluation in NHS clinical and hospice settings (and excluding higher education institution (HEI) settings), outcomes of Rounds attendance included a halving of significant psychological distress (General Health Questionnaire-12 > 3) in regular Rounds attenders, versus little change in non-attenders over the same period [18]. Rounds attenders reported improved empathy, communication and compassion for self, colleagues and patients, and improvements to clinical practice following Rounds (e.g. revision of protocols) [18]. The evaluation resulted in a realist evidence-informed programme theory describing how Rounds work in clinical settings, for whom, in what circumstances, and why.

While many of those ways Rounds work may transfer to HEI settings, there are significant contextual differences that may lead to altered or alternative outcomes for students, such as students having limited clinical experience. At the time of writing, Rounds had been implemented in approximately 21 UK higher education institutions licensed through POCF. Although there are some single-site, descriptive HEI evaluations [23,24], questions remain for how Rounds work in this setting and can be optimised, to benefit students and those they care for.

## Research questions

1. What do the available literature sources, augmented by realist interviews with key informants, offer to inform a programme theory of Schwartz Rounds in undergraduate settings: what works, for whom, in what circumstances, and why?
  - What outcomes do Rounds offer at personal, group and organisational levels for students, staff, placement providers and HEIs?
  - How do Rounds work (i.e. what are the key generative causal mechanisms that exist at personal, group and organisational levels) to produce those outcomes?
  - Which contextual factors significantly impact on how Rounds work?

## Methods

### Realist review

We approached the research questions using realist methodology in the form of realist review, being the first phase of our work, to be followed in future by a realist evaluation. It was important to conduct this preliminary work as a realist review to create programme theory (see below) that could be further developed, tested and refined in the subsequent evaluation phase, while enabling comparison to results from a previous realist evaluation of Rounds [18].

Realist review differs from other review methods in that it allows us to not merely ask “What happened?” but also explore context-sensitive questions such as “How, or why?” and “For whom, in what circumstances?” [25,26].

Realist inquiry considers our reality to have both an empirical, directly observable layer, as well as deeper “hidden” ontological layers that are considered to be as “real”, while not being directly observable. Realist analysis therefore attempts to uncover these hidden layers of reality, to capture the complexity of an intervention and the impact of contextual factors (individual and environmental) on how, why and for whom the intervention works.

The approach is underpinned by the assumption that interventions (in this case Schwartz Rounds) have an underlying intent and logic about how they are believed to “work” (for whom, in what circumstances, and why), which is often implicit or hidden. The researchers’ goal is to unveil this “programme theory” of the intervention through analysis and synthesis of the evidence. This is achieved by configuring theoretical elements with and against each other, using five techniques, summarised from Pawson [27] as follows: -

*Juxtaposing*: data from one source is used to augment another source, e.g. an outcome in one study is explained by a mechanism identified in another. *Reconciling*: contradictions between sources are resolved by analysis of contextual or implementation differences. *Adjudicating*: contradictions between sources are resolved by weighing the virtues and shortcomings of source methods/methodology. *Consolidating*: successful impacts of an intervention, attributed to different (rival) contexts and mechanisms across sources, are brought together into a fuller, multifaceted explanation. *Situating*: competing (rival) explanations are resolved by disambiguating the linked contexts and mechanisms, so that both explanations survive independently.

Therefore, realist review has a different focus than traditional systematic review. Theoretical components of an intervention are as likely to be found in a paper’s introduction or discussion sections as the results, or further afield in grey literature or public debates [28].

We developed, tested and refined the programme theory using the analytical heuristic of context-mechanism-outcome configurations (CMOs). Realist researchers consider an intervention’s outcomes (O) to be a product of generative underlying mechanisms (M), which are activated (or not) to varying degrees (a “dimmer switch”) within significant contextual factors (C): C + M→O [29]. Mechanisms can be conceptualised in various ways [30]; for this review we adopted the “resource plus response/reasoning” form [31], where mechanisms consist of both a resource associated with the intervention (e.g. free food and drink), coupled with the response or reasoning of those exposed to that resource (e.g. acceptance and gratitude).

### Review progression

The realist review followed the key steps outlined by Pawson et al. [25] and RAMESES guidance [26]: clarifying scope and identifying rough initial programme theory; searching for evidence; appraising evidence and extracting data; synthesising evidence and drawing conclusions.

A flowchart overview of the review stages and the progression through each article and interview can be found in supplementary file 1 (figure 2).

### 1 – Clarifying scope; identifying rough initial programme theory

The initial scope of inquiry was clarified and constrained to enable timely production of a programme theory, so that could serve as the starting point for our subsequent realist evaluation. Firstly, a rough initial programme theory was needed to “sketch the terrain” [26]. We debated incorporating the CMOs from Maben et al. [18] but ultimately chose to start with a blank slate. We preferred this open approach because we expected contextual differences between clinical and educational settings to be significant, such as students being especially disempowered, vulnerable, having lower clinical experience and confidence, and educators likely to be drawn into explicit teaching within Rounds (among other differences).

We identified Gishen et al. [23] as reporting the earliest UK HEI Rounds pilots (see Table 1, below). Gishen et al. [23] was therefore analysed abductively (i.e. induction to the most plausible explanation/“best fit”) and deductively to sketch out the rough initial contexts, mechanisms and outcomes and their (partial) configurations.

**Table 1:** Characteristics of included sources.


AUTHORS	SETTING	DESIGN	SUMMARY OF FINDINGS/DISCUSSION

Corless et al. (2009) [37]	USA – multidisciplinary educational	Evaluation of educational Schwartz Rounds over 4 years. Students surveyed post-Round (11 Rounds total) using 5-point Likert scale.	Students generally rated the Rounds highly – 86% of total evaluations were “excellent” or “exceptional”. 67% of students intended to attend future Rounds. Students valued discussions about communication with patients, families and other members of the multidisciplinary team.

Barker et al. (2016) [6]	UK – unidisciplinary (medical) educational	Discussion paper, drawing on qualitative data from one focus group, after two pilot Rounds.	The value of Rounds for students is linked to wellbeing, stress and burnout, with suggested benefits being a normalising of emotions and creation of channels for more open modes of communication.

Gishen et al. (2016) [23]	UK – unidisciplinary (medical) educational	Mixed-methods evaluation of 2 pilot Schwartz Rounds. Students surveyed post-Round (2 Rounds total) with rating scales and free-text comments. Student focus group (*n* = 7) explored views on one of the Rounds.	Mean Round scores (1 = “poor”, 5 = “exceptional”) were 3.5 and 3.3 for year 5 and year 6 student Rounds, respectively. 80% intended to attend future Rounds. 64% believed Rounds should be integrated in the curriculum. Qualitative feedback indicated a large audience size was a concern for some students. Students appreciated the opportunity to reflect without it being a written assessment. Students reported an appreciation of hearing the “more human” side of senior clinicians’ experiences.

Stocker et al. (2018) [39]	UK – unidisciplinary (medical) educational	Evaluation of pilot Round survey feedback from year 2 medical students (*n* = 83)	90 + % of students indicated the Round had a positive effect on their professional identity, across five domains, with 26% to 50% of students identifying a learning need in each domain. Written feedback reported being able to see fellow students as more human, although engagement with discussion was affected by lack of clinical experience.

Clancy et al. (2019) [44]	UK – multidisciplinary educational	Interpretative phenomenological analysis of interviews with student Rounds attenders (*n* = 8)	Three themes identified: students may question if it is safe to share within Rounds; not feeling alone with experiences and emotions; having space and time to discuss professional cultures when that otherwise would not happen.

Zervos and Gishen (2019) [45]	UK – unidisciplinary (medical) educational	Discussion paper of Rounds for medical students	Argues for Rounds as a way to diversify reflective opportunities within a medical curriculum, ‘bringing human elements centre stage’, where typically biosciences would dominate.

Gleeson et al. (2019) [41]	UK – unidisciplinary (medical) clinical	Mixed-methods evaluation of three pilot medical student Rounds within a major teaching hospital	45/84 year 3 medical students attended pilot Rounds. Quantitative feedback was very positive (80 + %) over twelve categories. 38/42 students found group reflection in this style to be preferable to written reflection.

Jakimowicz and Maben (2020) [24]	Australia, UK – multidisciplinary educational	Discussion paper of Rounds for nursing and other healthcare students	Argues for Rounds to be in place to support students and teaching staff, particularly in light of Covid-19 pressures on healthcare professions. Reports very positive feedback on pilot Rounds at each HEI in the UK and Australia.

Samad (2020) [46]	UK – unidisciplinary (medical) clinical	Letter to the editor in response to Gleeson et al. [41]	Cautions that preference for Rounds over written reflection may have been in part due to lack of a requirement to participate actively and that student engagement with Rounds reflection may have therefore varied.

Smith et al. (2020) [40]	UK – unidisciplinary (medical) educational	Evaluation of pilot Round survey feedback from year 2 medical students (*n* = 83)	Survey responses showed general agreement with ten statements describing learning and personal improvement. Five themes were found in written feedback with a mix of positive and negative sentiments. Notably, some students did not value the Round or find the themes relevant. Others appreciated hearing others’ experiences and felt the Round promoted empathy.

Smith (2021) [47]	UK – multidisciplinary educational	Discussion paper of implementing student Rounds at author’s HEI	Discusses adaptation of Rounds to online delivery following restrictions due to Covid-19. Opportunities to reach students who might otherwise not be able to attend, but challenges with the technology.

Abnett et al. (2021) [42]	UK – unidisciplinary (medical) and multidisciplinary clinical	Mixed methods evaluation of one unidisciplinary and one multidisciplinary online Round for students in clinical practice	Student feedback was overall positive for the Rounds, with the unidisciplinary Round rated slightly higher. Some students expressed that well-prepared panellist stories were a barrier to participation in the audience phase, as it could be difficult to contribute at the same polished level.


### 2 – Searching for evidence

The unique name (and proprietary licensing) of the intervention simplified database searching by including all articles in English that included “schwartz” within five words of “round?” in the title or abstract (and full text, where available). Databases were chosen to cover academic and grey literature focusing on education and/or healthcare: British Nursing Index (BNI); Cumulative Index of Nursing and Allied Health Literature (CINAHL); Educational Resources Information Centre (ERIC); MEDLINE; OpenGrey (until its closure in 2020); Open Access Theses and Dissertations (OATD); and PsycINFO.

On reviewing the initial search results, further search constraints were found to be unnecessary, as no significantly off-topic papers were included.

Searching was periodically refreshed to capture newly published material (last search date: 03/11/2023), with concurrent monitoring of Twitter hashtags (#SchwartzRounds) and a Google Alerts query. Attempts were made to identify additional material by snowballing backwards (reviewing articles’ reference lists) and forwards (searching citations using Google Scholar).

### 3 – Appraising evidence and extracting data

Articles were included for analysis based on a judgement of “relevance and rigour”, following the guidance of Pawson [27] and Wong [32].

Reported studies and any other textual sources (editorials, letters, etc.) were included as relevant to developing, testing and refining programme theory, where they featured some theoretical discussion of Rounds run for pre-registration healthcare students (medical, nursing, midwifery and other allied health professions). Textual sources were excluded (for relevance) if they did not represent an intervention that followed core features of the Rounds model, as described in Taylor et al. [33] and Leamy et al. [21].

Rigour was assessed as per Wong [32] by questioning *coherence* (how does material fit with other existing evidence, theory or logic?) and *trustworthiness* (has data been produced with transparent and reliable methods?).

Extraction from included sources to the programme theory working document was conducted in two stages: first-pass note-taking, followed by drafting programme theory statements from the source.

First-pass note-taking (in Microsoft OneNote) involved a four-column table headed “IF (context) – THEN (mechanism) – SO (outcomes) – Comments”. This initial “practice” guided the subsequent drafting stage, where programme theory statements were extracted and arranged within the programme theory working document, grouping related elements and attempting to be faithful to source wording, where possible.

This process, as with all realist review, was non-linear, iterative and involved a range of abductive and deductive judgement to extract and synthesise CMOs. The process included regular presentation of the programme theory within the research team for discussion and challenge.

### 4 – Synthesising evidence and drawing conclusions

The extraction and analysis process, described above, was applied in turn to the twelve included textual sources (table 1; supplementary file – figure 2). Development, testing and refinement of the programme theory applied Pawson’s guidance for juxtaposing, adjudicating, reconciling, consolidating and situating the evidence [27] (see above). The working programme theory document was periodically redrafted, reorganised and refined, to maintain clarity and brevity.

### Realist interviews

As part of the iterative refinement of a realist review it is often recommended to explore the programme theory with stakeholders who have expert knowledge and/or experience. This helps develop, test and refine the programme theory, as well as ensure findings are useful to the end-user [26,27,34]. Accordingly, we also conducted realist interviews [31,35,36] with four key informants involved in introducing Rounds into undergraduate settings.

### Interview sampling

Participants were purposively sampled for maximum relevance and knowledge of Rounds in undergraduate settings, from publicly available information on individuals who were involved in the earliest adoption of Rounds within HEIs in the UK. We approached two experienced facilitators of student Rounds (LG, FG) and two staff from the Point of Care Foundation (JG, RB) who had assisted in establishing Rounds at those HEI sites. All participants gave informed consent to be identified, to aid in transparency – FG and RB were authors on included papers.

### Interview data collection

Interviews were conducted in-person (*n* = 1) or by telephone (*n* = 3), using a semi-structured topic guide (supplementary file 1), lasting approximately 40 minutes each. Questions were designed to develop, test and refine programme theory, following advice from Manzano [35] for content of phase 1 (“theory gleaning”) and phase 2 (“theory refinement”) interviews. Interviews took place following initial analysis of three textual sources to allow for testing and refinement of CMOs through a focused topic guide (all but one other textual source were published subsequent to our interviews).

### Interview analysis

Interviews were transcribed and analysed using the same process as for literature sources, i.e. identifying partial and full CMOs, developing, testing and refining the programme theory (see above). All included sources (articles and interviews) were periodically reappraised, to identify further contributions to CMOs in response to the iteratively refined programme theory.

### Ethics

This study was granted ethical approval by the University of Surrey Ethics Committee (ref. UEC 2019 028 FHMS, 3^rd^ May 2019).

## Results

### Search

Thirteen articles were identified for initial inclusion from database searching and citation snowballing, following brief full-text review for relevance and rigour [6,23,37,38,39]. One paper was subsequently excluded for departing from key features of the Rounds model: Shield et al. [38] described an intervention (“Schwartz Communication Sessions”) inspired by Schwartz Rounds but were a distinct intervention. Of the twelve included articles, seven were evaluations of student experiences of Rounds, while five were discussion articles (including editorials, letters) (table 1). One evaluation was from the US [37], one discussion article from Australia/UK [24], with the remainder UK-based. While Stocker et al. [39] and Smith et al. [40] reported features atypical for Rounds (mandatory attendance, small group discussion and feedback) we have included them to capture those contextual variations. We have also included two studies on Rounds for healthcare students (exclusively) that took place in clinical settings rather than within a HEI [41,42], as we assessed these to still be highly relevant.

Due to the search strategy, a large number of articles with theoretically thin mentions of Schwartz Rounds were found (e.g. Ho [43]), often within brief editorials and opinion pieces (276 articles excluded). These articles were nevertheless screened to confirm they did not contain insights relevant to the research questions. A PRISMA diagram is contained in supplementary file 1 (figure 1).

### Programme theory

The finalised programme theory, expressed as CMOs, is presented in tables 2 and 3 in ten thematic groupings. Five “core” CMO groups are in table 2 (essential elements of the Rounds model), with five “contextual adaptations” CMO groups (modulating effectiveness, without being a core feature) in table 3. Individual CMOs are stated as closely as possible in the format *context* + *mechanism (resource-reasoning/response)* → *outcome*. A version annotated with Cs, Ms and Os is included in supplementary file 1, but these annotations are removed below to maintain readability. Please note, the group headings are summary labels, not CMOs. Some CMOs may appear incomplete, e.g. where mechanisms were difficult to identify.

**Table 2:** Core CMO groups.


**1 – Rounds provide a reflective space to discuss emotional challenges, thereby enhancing insight and wellbeing** When discussion of difficult topics (e.g. death and disease) in professional care-giving is culturally taboo, and when HEIs and students alike focus on teaching and assessment of technical knowledge and skills over social and emotional aspects of care work, then a curriculum gap is created around the human side of caring. When students are exposed to the challenges of clinical placements during their study, then Rounds provide a safe and containing space for reflection on the nature of the work and emotional challenges, to fill the curriculum gap, preparing students for the stresses and demands of professional care work, contributing to them being better able to manage their emotions and be resilient, improving student wellbeing. When reflection, insight and wellbeing is enhanced, then students can successfully maintain empathetic, compassionate practice, in part due to a reduction in (or absence of) the symptoms of burnout. This can improve healthcare professional (HCP) and service-user experiences of care-giving, strengthen communication with both HCPs and service-users, reduce errors attributable to stress, reduce sickness absence and potentially increase retention.

**2 – Rounds promote an open and humanised professional culture, enhancing connectedness with colleagues and patients** When professional standards are so high that it is almost impossible to meet those ideals under real-world constraints, then experienced HCPs sharing their experiences of vulnerability can recontextualise the meaning of professionalism for students, leading to them experiencing less of a distressing disconnect when exposed to practice. When feeling challenged by their work (including early-career imposter syndrome), this allows students to judge themselves less harshly against high professional standards, and realise many professionals experience similar feelings (but mask them well). While Rounds in clinical settings are not explicitly for learning, when Rounds are used in undergraduate settings, the educational setting may make some learning goals more explicit, such as teaching students about reflection, vulnerability, compassionate care, inter-professional relationships, owning up to mistakes, and showing a more human side. With skilled facilitation which focuses discussion on the impact on the practitioner (not problem solving), such teaching may be achieved without being judgemental or didactic. Open discussion of emotional and social challenges within Rounds can help students realise the benefits of talking things through and being listened to, replacing unhealthy/dysfunctional coping styles (e.g. emotional distancing, alcohol use) with healthy coping behaviours. Students learn to acknowledge and process emotions rather than suppress them, and learn the value of peer-support, promoting connectedness with colleagues. Open discussion of emotional and social challenges can also benefit students’ learning of advanced communication skills in those areas, blending their personal humanity into professional conversations.

**3 – Rounds offer role-modelling of vulnerability which enables greater interpersonal connectedness** When the expression of emotion is culturally suppressed and when panellist stories are from senior clinicians, students observe role-modelling of counter-cultural behaviours (openness, vulnerability, courage to have difficult conversations), which offers permission to students to adopt those behaviours, including enabling students to share their own stories, within and outside of Rounds. More experienced colleagues are humanised, appearing more approachable and less intimidating, breaking down barriers to communication and bringing HCPs into closer working relationships. When students share their own stories, in a Round that focuses on students, that disrupts the established hierarchies with teachers and clinicians, allowing students to be humanised and not just seen as vessels to be filled with teaching. If a panellist is particularly senior or experienced, students’ opportunity to hear them talk vulnerably and candidly are rare or non-existent. Students’ cultural expectations are therefore subverted, enhancing the impact of the story. Similarly, student panellist stories may be less subversive. However, when Rounds includes stories from many levels of experience and seniority, then a more complete range of perspectives paints a fuller picture.

**4 – Rounds are impactful when facilitators focus panellists and audience members on emotional and relational elements** If panellists are prepared well, with time dedicated to each panellist by the facilitator to help identify elements of their story most beneficial to a Round, ideally with the other panellists also present, then Rounds are more effective, needing less active steering from the facilitator to focus discussion on what the Round is for. However, when panellists are well-prepared then students may feel less confident in following and therefore may decide not to speak. If panellists are not well-prepared, then their story may be too clinically technical, or lack closure. When a student audience desires that closure (reportedly more so than experienced/qualified staff), then time is spent asking questions of the panellists to provide that closure, rather than reflection on the human dimensions of the story.

**5 – Rounds offer reflective insights from a wide range of perspectives** If panellist stories are drawn from multiple disciplines, then students are exposed to a range of perspectives on a case or theme, giving students greater insight on HCP and service-user experiences, that goes beyond students’ individual experiences. Group reflection promotes compassion and empathy among students, with the examination of past experiences providing a guide for future events. When teachers or clinicians are present, discussion in Rounds generates insights for them, in addition to students.


**Table 3:** Contextual adaptations CMO groups.


**6 – Rounds allow reflection to be more engaging for students when they are non-mandatory** When Rounds are included as part of the curriculum, mandatory attendance should not be considered long-term, as the style of reflection will not suit all students and, through losing an important core feature of Rounds (voluntary attendance), the nature and tone of Rounds may be detrimentally changed. However, when Rounds are complex and countercultural, initially mandating students attend a small number of Rounds could help students understand their purpose and value, increasing attendance from students who will find Rounds valuable. If Rounds are non-mandatory, then students can engage more enthusiastically with reflection, as compared to mandatory written reflections that are typical of HCP training, leading to different insights and making the reflection more effective. However, when Rounds are a non-mandatory, non-assessed activity, students may not immediately understand their value, requiring some time to adapt to and accept. When opportunities for reflection are limited within a curriculum, if Rounds are scheduled as part of curricular time, then a larger scale, confidential forum for reflection is available to students, creating more opportunities for students to participate in reflection.

**7 – Perceptions of safety within a Round varies based on multiple factors** When the audience size of in-person Rounds is not so large as to be intimidating for students to share personal stories (35 to 50 people), then audience participation is likely to be higher, increasing disclosure from students and therefore enabling reflective insights. At this audience size, everyone present can see and sense each other comfortably, increasing the sense of safety and allowing for facilitators to be more effective, both during and after the Round. Without this limit on audience size, discussions may happen instead in post-round ad hoc conversations, within smaller peer groups, without the safety of professional facilitation. If the audience is multidisciplinary, then students are exposed to a broader range of perspectives and recognise experiences in common, promoting connectedness and understanding of interprofessional working. If the audience is unidisciplinary, then having a range of panellists from different professions can still provide a valuable range of perspectives. If the audience is unidisciplinary, then they may feel freer or more confident to speak up and contribute their own story to the Round. When Rounds are run solely for a student audience, then the reduced perception of hierarchies found in clinical practice allows students to have courage to make their voices heard, creating a safer space to share thoughts with confidence, compared to clinical Rounds where students can feel less “safe” to speak. Student confidence to share depends on a sense of safety and containment, which can be affected by clinical experience, a younger age, the pace of conversation, willingness to tolerate emotional discomfort, and fear of judgement from peers and teaching staff.

**8 – Adapting timing and themes to students’ changing needs may improve engagement** Over a course of study, students’ interests, short term priorities (such as assessments) and level of clinical experience varies. When this context is taken into account, Rounds themes and timing within the curriculum can be tailored to students’ interests and needs, improving engagement and attendance.

**9 – Resonance with Rounds’ stories is affected by clinical experience levels** When students lack clinical experience, then they may not have acquired the contextualised knowledge to be able to respond as meaningfully to panellist and audience stories, reducing the value of that reflection. As students acquire clinical experience, that experience increases the resonance of Rounds stories, as well as increasing students’ need for the benefits of Rounds. However, when Rounds are trying to change the culture of conversations, and when students already feel pressured by judgement and scrutiny, then introducing Rounds as early as possible (e.g. inclusion of first-years) allows more time for cultural change to happen. When lack of clinical experience may be a limiting factor, then using a more generic theme or one related to student experience can still enable students to engage in relationally focused conversations, and consequently benefit from them. When students qualify and gain more clinical experience of emotional and relational challenges, they can apply insight from Rounds attended as an undergraduate, revealing new understanding of stories upon applying the reflective learning in practice.

**10 – Recorded or online Rounds can increase reach but presents risks for psychological safety** Through video recording and sharing Rounds online, further benefits can be realised such as distance learning and reincorporation of material into the taught curriculum, expanding access to Rounds’ insights. However, the act of recording may inhibit the confidentiality and perceived safety of the Round, reducing attendees’ willingness to share their stories, or alter the openness and honesty with which they do so. When a Round is not experienced in-person, then auditory and visual cues (body language, facial expressions) from others cannot be easily experienced, inhibiting emotional connection and understanding. If the Round experience is online, that lack of direct contact can reduce awareness and opportunity for facilitators to follow up with anyone who may be especially distressed by the Round discussion, meaning extra “safety net” processes may be necessary.


#### 1 – Rounds provide a reflective space to discuss emotional challenges, thereby enhancing insight and wellbeing

This group provides an outline rationale for Rounds as an intervention for undergraduates. Articles and interviewees were overall very consistent in describing the role of Rounds and reasoning for them in healthcare education. Barker et al. [6] and Jakimowicz and Maben [24] supported this further, by reference to primary evidence in areas such as staff wellbeing and patient experience. Corless et al. [37] differed slightly by positioning Rounds more explicitly as a communication skills intervention, while Clancy et al. [44] emphasized the opportunities for interprofessional learning. Despite several more years of experiences running Rounds in HEIs, there was consistency between pilot evaluation papers and later interview responses on what Rounds were “for” and could achieve: -

“I think we’re still not there at all. I think there is still a huge curriculum gap in terms of giving people confidence about the importance of those relational skills and paying more attention to them. […] actually I think they build relational skills, through things like a Schwartz Round, in a very different way to other ways of learning.” – JG

Student interviews in Clancy et al. [44] confirmed that students experienced Rounds as an opportunity to talk about important aspects of healthcare work that otherwise is not given space and time.

#### 2 – Rounds promote an open and humanised professional culture, enhancing connectedness with colleagues and patients

Articles and interviewees highlighted that there were unhealthy cultures of competitiveness and lack of open conversations about experiencing challenges or making mistakes, particularly in medical schools/practice. Rounds were seen as a way to have those conversations, so that students learned within a culture that demonstrated valuing open and honest connection with colleagues and patients: -

“Medical education is no longer about just teaching core scientific skills and knowledge, it’s about professional behaviours, it’s about professional attitudes, it’s about personal wellbeing, it’s about self care. […] So Schwartz Rounds are a tool in an arsenal of… skills, attitudes, behaviours, that will hopefully enable our future doctors to look after patients better, but also look after themselves better.” – FG

Barker et al. [6] also suggested a role for Rounds as being therapeutic, through beneficial effects of group sharing and listening in a non-judgemental space. Thus, for students, Rounds can both teach and enable empathetic communication. Stocker et al. [39] demonstrated in student comments how learning outcomes and problem solving can become more explicit within HEI Rounds.

#### 3 – Rounds offer role-modelling of vulnerability which enables reater interpersonal connectedness

“They love to learn from those ‘powerful’ role models, clinician role models. […] people were having their stereotypes and myths exploded in front of their eyes. There was somebody very ‘powerful’ who was showing their vulnerability and sadness at the death of a lonely patient.” – FG

Although more junior perspectives were also considered valuable: -

“I think it is really important sometimes to have very senior people role modelling those different types of emotions. Equally I think it’s really good sometimes to have students, talking about how they compare themselves to other students, or their own particular fears, or early encounters that students remember.” – JG

There was considerable support from student interviews in Clancy et al. [44] for how Rounds had humanised colleagues while reducing interprofessional boundaries.

#### 4 – Rounds are impactful when facilitators focus panellists and audience members on emotional and relational elements

Articles and interviewees highlighted the need for panellists to have well-prepared, coherent stories (through facilitator guidance) to build a narrative that fits the Round’s focus on emotional and relational aspects of care-giving.

“So when I prepare people I write down almost a script for them, it’s not written in prose but it’s got bullet points and then I go through and highlight the Schwartzy themes, and I highlight the reflection and I break down quite didactically for people, how they introduce themselves, what the story should look like […] There’s often a very visceral event, that’s kind of “haunted” the panellist. So I break it down very carefully.” – FG

Corless et al. [37] reported one session that received noticeably poorer feedback for the panellists’ presentation being too focused on technical content (perhaps being less well-prepared by facilitators). Abnett et al. [42] reported some students found polished panellist stories to be intimidating to follow when discussion was handed over to the audience.

#### 5 – Rounds offer reflective insights from a wide range of perspectives

Articles and interviewees considered Rounds to be useful to students through exposing them to a range of experiences and perspectives beyond their own, including from other healthcare disciplines (both through panellists and others in the audience): -

“Well I think the value in practice is hearing about a patient or a situation from different points of view. So […] let’s say you’ve got a doctor, a therapist, occupational health or physio or psychology, and say a ward clerk or a security guard or a porter, like you might have in a hospital Round. That would be incredibly valued, valuable.” – RB

Student feedback in Abnett et al. [42] confirmed that students recognised the benefits from interprofessional learning, gaining greater understanding of other students’ roles.

#### 6 – Rounds allow reflection to be more engaging for students when they are non-mandatory

Interview participants identified tensions around requiring students to attend Rounds. The value of Rounds was not always immediately apparent to students, requiring attendance to fully understand. However, students were known to dislike mandatory reflection: -

Interviewer (DH): “Is that the danger then, if having it within a curriculum, if it was made mandatory is that people would just rebel against that just for the sake of you being told to do it?”

RB: “I think it would be helpful if it was mandatory just to attend one session – so all students have an idea of what a Schwartz Round it. After that the decision of whether or not they attend should be voluntary.”

This was tested in Smith et al. [40] where year 2 medical students attended a mandatory Round, student feedback suggested some students did not feel the relevance of Rounds discussion to their education/practice, with several students expressing that forced group reflection had been perceived as a facilitated complaints session.

Interviewees were clear that Rounds provided an additional, different reflective opportunity to mandatory, written reflection activities: -

“So there are lots that we knew medical students wanted to talk about, but they didn’t have a confidential… space, that looked like a Schwartz Round to talk in. They had some reflective opportunities, but our students had told us that they didn’t like written reflection and they found it ‘rude’ that we marked it and graded it.” – FG

This was confirmed in Gleeson et al. [41] with year 3 medical students overwhelmingly preferring group reflection in Rounds to written reflection activities, although Samad [46] cautioned that this was a self-selected group, excluding the 47% of medical students who chose not to attend. Students were similarly supportive of using multiple reflection modalities in Abnett et al. [42], while expressing dislike of mandatory written reflections.

#### 7 – Perceptions of safety within a Round varies based on multiple factors

Articles and interviewees revealed that educational Rounds could grow to sizes not commonly seen in other settings and based on student feedback (Gishen et al. [23] and Gleeson et al. [41]) this could compromise psychological safety, as audiences grew larger: -

“[…] the consensus view is 50 feels about right. Because it allows a kind of conversation, and allows us as facilitators and panellists to be able to see everybody, and everybody be able to see everybody as well. So our preference has been that size. […] It feels more containing really, we can see the faces of the students and see who looks particularly stressed […]. So a bit safer, maybe that’s the word I’m trying to get to. And there is a downside in that there’s a lot of enthusiasm for the Rounds, means we get fewer students, but it feels more, sort of more emotionally containing and safe.” – LG

Interviewees mostly agreed that a multidisciplinary audience was preferable, to provide a range of views, but not always practical at medical schools. A student Round was thought to be more comfortable for students than attending Rounds in clinical settings: -

“[…] it really struck me how students, particularly medical students, really have that hierarchical model of a hospital drummed into them – I imagine it’s the same for nurses as well – and often therefore they’re very hesitant about having the courage to make their voices heard, to say what they’re thinking, and so I think probably having a forum just for them makes sense in terms of giving them the confidence, and making it valid to put in their own point of view. More than they would if it were a much more mixed audience.” – JG

Clancy et al. [44] reported on a number of factors affecting students’ perceptions of safety within a Round, revealing that students did not always trust peers and teaching staff would withhold judgement. This fear of judgement was echoed by students in Abnett et al. [42].

#### 8 – Adapting timing and themes to students’ changing needs may improve engagement

Articles and interviewees highlighted that students were not a homogenous group and their interests, priorities and needs would vary over their course of study. Gishen et al. [23] reported imminent exams as a potential barrier to Rounds attendance, while Zervos and Gishen [45] discussed successfully tailoring Rounds for each year group. Keeping Rounds themes generic was thought to help broaden their appeal: -

“[…] we tend to go for more generic topics like *In At The Deep End, What I Learned From A Patient*, things like that we’ve found work best, because [students] are in training and working across a range of settings, but that’s fine. And also mindful of the stage that they’re at, there is a limit to their experience, as well.” – LG

#### 9 – Resonance with Rounds’ stories is affected by clinical experience levels

Interviewees suggested that both the students’ benefit of (and need for) Rounds was at least partly linked to clinical experience.

“They’re mainly all year three, we aim that at year three because we felt the undergraduate programmes needed to have enough clinical experience to benefit from.” – LG

Interviewer (DH): “So you think that that clinical experience is, is it useful, or is it more towards the end of necessary for Rounds to be having an effect?”

RB: “Well I think it would have more resonance if they’d already started being with patients, or about to start. And I also think that they would need it, yeah they would need it more. And they would see the need.”

Stocker et al. [39] included student feedback suggesting that the impact of a Round for a year 2 medical student might be reduced due to a lack of clinical experience. A counter-point to this was an argument from Stocker et al. [39], Abnett et al. [42] and JG, for Rounds benefitting from early opportunities to take effect.

“I think the earlier you run Rounds the better to be honest, because I think… getting the benefit from the Rounds, you get most benefit when you’re able to enter into that kind of honest, transparent discussion where you can connect your own emotions with whatever it is that you’re talking about. And that can relate to you know, very simple aspects about studying, about relationships with other students, about relationships with teachers.” – JG

#### 10 – Recorded or online Rounds can increase reach but presents risks for psychological safety

Corless et al. [37] briefly reported the recording and sharing of Rounds through a virtual learning environment and this idea was further explored with interview participants, who were open to the idea but hesitant about risks: -

“I’d have to think through that pretty carefully? […] And actually, it’s one thing filming the panel, who are generally- I mean I have used students on panels before but the panels are generally seasoned clinicians and other healthcare workers. I feel […] I have a real responsibility to protect students, and… I would have to think very carefully about how I, in the future, would, could, should, approach the issue of using their reflections – that they may consent to as twenty-year-olds – but may not want forever available, for the rest of their careers. I don’t know, I think that’s fraught with ‘danger’.” – FG

As Rounds began to be held online with Microsoft Teams, Smith [47] and Abnett et al. [42] were able to point to some challenges with the technology, particularly with respect to maintaining a sense of cohesive space and feeling connected when many cameras might be turned off.

## Discussion

### Summary

This realist review has uniquely produced an evidence-informed programme theory of Rounds in undergraduate settings. As well as identifying core features of how Rounds work in this setting (as distinct to clinical settings), it has also identified areas of adaptation that may impact on Rounds’ effectiveness, such as mandatory attendance. This is an important and timely contribution, given the focus on healthcare student retention and wellbeing, and the rapid expansion of Rounds in HEIs in the UK and beyond.

### Comparison to other existing evidence

Maben et al. [18] reported a final evidence-informed theory of Rounds in healthcare settings with nine key CMOs: trust, emotional safety and containment; group interaction; countercultural/third space for staff; storytelling; role-modelling vulnerability; shining a light on hidden stories/roles; self-disclosure; contextualising patients and staff; reflection and resonance.

There are some clear similarities and differences to our CMOs synthesised for this review. While our “core” CMO groups overlap with Maben et al. [18] they also differ for the educational context with a greater emphasis on filling a “curriculum gap” and enacting cultural change from an early stage. It was apparent from multiple sources that educators and students alike felt that Rounds should be introduced as early as possible to realise the benefits found in our “core” CMO groupings and Maben et al. [18].

Of note, “group interaction” might be weaker in student Rounds, considering the often highly varied audience in other settings (many disciplines, roles, levels of experience and seniority), particularly if a student Round audience is not multidisciplinary. Similarly, “shining a light on hidden roles” may be impacted if student Rounds facilitators do not have immediate access to a range of HCPs to recruit as panellists, or if the student audience is not multidisciplinary. While HCPs rarely have much opportunity to prioritise time for reflection and emotional discussion outside of regulatory requirements (e.g. revalidation), students are typically offered (even mandated) reflective activities throughout their study, which may affect how Rounds are perceived, both positively or negatively.

Our “contextual adaptations” CMO groups highlight clearly that for Rounds to be adapted successfully from the clinical model, the various and varying preferences and needs of students must be taken into account, with differing priorities (e.g. academic performance), clinical experience and confidence levels.

With global healthcare worker shortages estimated to be up to 10 million by 2030 [48], student wellbeing is critically important to current and future healthcare delivery, with Rounds existing alongside a range of wellbeing and reflection initiatives. Although sharing some features with Balint groups, Rounds have a unique combination of features that combine to produce their outcomes [33]. Some HEIs are therefore running both Balint groups and Rounds to provide a range of opportunities to suit students’ varying preferences [7]. Rounds are inclusive, “for everyone”, attended as-needed, whereas Balint groups originated in primary care (for GPs) and have typically targeted medical students, with a fixed group membership. Variance in availability is found with other support interventions, such as restorative clinical supervision, peer-buddying or informal coffee-morning drop-ins. The RePAIR report [17] found a range of support in place for English healthcare students and further innovative examples can be found in the literature, such as a SMS text support service [49] and online models like the Big White Wall [50]. Rounds are structured to provide a safe space to have conversations that wouldn’t or couldn’t happen elsewhere, making Rounds compelling to add alongside existing reflection and wellbeing interventions, rather than competing with them.

## Strengths and limitations

This is the first published review of Rounds specifically in healthcare undergraduate settings and although it follows a systematic review of Rounds studies [33] and a realist-informed evaluation [18], it is also the first realist review of Rounds literature. This facilitates easier integration and comparison with the previous realist evidence [18] and provides an important starting point for our subsequent realist evaluation (in progress). The synthesis of textual sources was supplemented with interviews with key informants with deep knowledge of student Rounds at the HEIs where they have run the longest. These insights are highly valuable for an intervention that continues to experience rapid uptake in the UK and beyond.

Textual sources were initially limited by Gishen et al. [23] and Barker et al. [6] sharing authors and qualitative data sources, with two authors from these papers included as interview participants for this review. This was attributable to the (initially) low number of HEI sites that had implemented Rounds, six at the time of interviewing, however subsequently published textual sources included are more diverse, albeit with some remaining overlap (e.g. Stocker et al. [39], Smith et al. [40]).

## Conclusion

Schwartz Rounds are a unique intervention that can support healthcare students through their pre-registration education and may enhance patient care by developing a greater sense of connectedness and insight. The five “core” and five “contextual adaptation” CMO groups presented can serve as organisational guidance for HEIs running or in the process of setting up Rounds for their undergraduates, identifying important considerations for implementation. A subsequent realist evaluation is underway to test and expand on this programme theory.

## Additional File

The additional file for this article can be found as follows:

10.5334/pme.930.s1 Supplementary File 1. PRISMA diagram, review progression flowchart, annotated (C, M, O) programme theory tables, table of abbreviations, and interview topic guide.

## References

[1] Groothuizen JE, Callwood A, Allan HT. The ‘values journey’ of nursing and midwifery students selected using multiple mini interviews: Evaluations from a longitudinal study. Nurs Inq. 2019; 0: e12307. DOI: 10.1111/nin.12307 31240793

[2] Higher Education Policy Institute. The invisible problem? Improving students’ mental health. Oxford: Higher Education Policy Institute; 2016. Retrieved from http://www.hepi.ac.uk/2016/09/22/3592/.

[3] Sheldon E, Simmonds-Buckley M, Bone C, et al. Prevalence and risk factors for mental health problems in university undergraduate students: A systematic review with meta-analysis. J Affect Disord. 2021; 287: 282–92. DOI: 10.1016/j.jad.2021.03.054 33812241

[4] Li Y, Wang X, Zhu X, Zhu Y, Sun J. Effectiveness of problem-based learning on the professional communication competencies of nursing students and nurses: A systematic review. Nurse Educ Pract. 2019; 37: 45–55. DOI: 10.1016/j.nepr.2019.04.015 31082712

[5] Chan KD, Humphreys L, Mey A, et al. Beyond communication training: The MaRIS model for developing medical students’ human capabilities and personal resilience. Med Teach. 2019; 1–9. DOI: 10.1080/0142159X.2019.1670340 31608726

[6] Barker R, Cornwell J, Gishen F. Introducing compassion into the education of health care professionals; can Schwartz Rounds help? J Compassionate Health Care. 2016; 3: 3. DOI: 10.1186/s40639-016-0020-0

[7] Gishen F, Zervos M. Between a doc and a hard case: a journey through liminality. The BMJ; 2018. https://blogs.bmj.com/bmj/2018/09/07/between-a-doc-and-a-hard-case-a-journey-through-liminality/ (accessed October 13 2019).

[8] Australian Nursing & Midwifery Accreditation Council. Registered Nurse Accreditation Standards 2019. Canberra: Australian Nursing & Midwifery Accreditation Council; 2019. Retrieved from https://www.anmac.org.au/standards-and-review/registered-nurse.

[9] Nursing and Midwifery Council. Standards for pre-registration nursing programmes. London: Nursing and Midwifery Council; 2023. Retrieved from https://www.nmc.org.uk/standards/standards-for-nurses/standards-for-pre-registration-nursing-programmes/.

[10] Health Education England. Values Based Recruitment Framework. London: Health Education England; 2016. Retrieved from https://www.hee.nhs.uk/our-work/values-based-recruitment.

[11] Neumann M, Edelhäuser F, Tauschel D, et al. Empathy Decline and Its Reasons: A Systematic Review of Studies With Medical Students and Residents. Acad Med. 2011; 86: 996. DOI: 10.1097/ACM.0b013e318221e615 21670661

[12] Chen DCR, Kirshenbaum DS, Yan J, Kirshenbaum E, Aseltine RH. Characterizing changes in student empathy throughout medical school. Med Teach. 2012; 34: 305–11. DOI: 10.3109/0142159X.2012.644600 22455699

[13] Maben J, Latter S, Clark JM. The sustainability of ideals, values and the nursing mandate: evidence from a longitudinal qualitative study. Nurs Inq. 2007; 14: 99–113. DOI: 10.1111/j.1440-1800.2007.00357.x 17518822

[14] Curtis K, Horton K, Smith P. Student nurse socialisation in compassionate practice: A Grounded Theory study. Nurse Educ Today. 2012; 32: 790–5. DOI: 10.1016/j.nedt.2012.04.012 22583813

[15] Gishen F. Suicide among medical students. BMJ. 2019; 366: l5465. DOI: 10.1136/bmj.l5465 31501160

[16] Alshahrani Y, Cusack L, Rasmussen P. Undergraduate nursing students’ strategies for coping with their first clinical placement: Descriptive survey study. Nurse Educ Today. 2018; 69: 104–8. DOI: 10.1016/j.nedt.2018.07.005 30029041

[17] Health Education England. Reducing Pre-registration Attrition and Improving Retention. London: Health Education England; 2018. Retrieved from https://www.hee.nhs.uk/our-work/reducing-pre-registration-attrition-improving-retention.

[18] Maben J, Taylor C, Dawson J, et al. A realist informed mixed methods evaluation of Schwartz Center Rounds® in England. Health Serv Deliv Res. 2018; 6. DOI: 10.3310/hsdr06370 30431750

[19] Robert G, Philippou J, Leamy M, et al. Exploring the adoption of Schwartz Center Rounds as an organisational innovation to improve staff well-being in England, 2009–2015. BMJ Open. 2017; 7: e014326. DOI: 10.1136/bmjopen-2016-014326 PMC5223680 28057662

[20] Schwartz KB. A patient’s story. BostonGlobe.Com. (14 July 2012). https://www.bostonglobe.com/magazine/1995/07/16/patient-story/q8ihHg8LfyinPA25Tg5JRN/story.html (accessed 26 November 2023).

[21] Leamy M, Reynolds E, Robert G, Taylor C, Maben J. The origins and implementation of an intervention to support healthcare staff to deliver compassionate care: exploring fidelity and adaptation in the transfer of Schwartz Center Rounds® from the United States to the United Kingdom. BMC Health Serv Res. 2019; 19: 457. DOI: 10.1186/s12913-019-4311-y 31286958 PMC6615238

[22] Reed E, Cullen A, Gannon C, Knight A, Todd J. Use of Schwartz Centre Rounds in a UK hospice: Findings from a longitudinal evaluation. J Interprof Care. 2015; 29: 365–6. DOI: 10.3109/13561820.2014.983594 25421453

[23] Gishen F, Whitman S, Gill D, Barker R, Walker S. Schwartz Centre Rounds: a new initiative in the undergraduate curriculum—what do medical students think? BMC Med Educ. 2016; 16: 246. DOI: 10.1186/s12909-016-0762-6 27658411 PMC5034622

[24] Jakimowicz S, Maben J. “I can’t stop thinking about it”: Schwartz Rounds® an intervention to support students and higher education staff with emotional, social and ethical experiences at work. J Clin Nurs. 2020; 29: 4421–4424. DOI: 10.1111/jocn.15354 32472584

[25] Pawson R, Greenhalgh T, Harvey G, Walshe K. Realist review-a new method of systematic review designed for complex policy interventions. J Health Serv Res Policy. 2005; 10: 21–34. DOI: 10.1258/1355819054308530 16053581

[26] Wong G, Westhorp G, Pawson R, Greenhalgh T. Realist Synthesis: RAMESES Training Materials; 2013. http://www.ramesesproject.org/media/Realist_reviews_training_materials.pdf (accessed 26 November 2023).

[27] Pawson R. Evidence-Based Policy: A Realist Perspective. London; SAGE Publications Ltd; 2006. DOI: 10.4135/9781849209120

[28] Pawson R, Owen L, Wong G. The Today Programme’s Contribution to Evidence-based Policy. Evaluation. 2010; 16: 211–3. DOI: 10.1177/1356389010369636

[29] Dalkin SM, Greenhalgh J, Jones D, Cunningham B, Lhussier M. What’s in a mechanism? Development of a key concept in realist evaluation. Implement Sci. 2015; 10: 49. DOI: 10.1186/s13012-015-0237-x 25885787 PMC4408605

[30] Westhorp G. Understanding mechanisms in realist evaluation and research. In Emmel N, Greenhalgh J, Manzano A, Monaghan M, Dalkin S (eds.), Doing Realist Research. Los Angeles; SAGE Publications Ltd. 2018. pp. 41–58. DOI: 10.4135/9781526451729.n4

[31] Pawson R, Tilley N. Realistic Evaluation. London; SAGE Publications Ltd; 1997.

[32] Wong G. Data Gathering in Realist Reviews: Looking for needles in haystacks. In Emmel N, Greenhalgh J, Manzano A, Monaghan M, Dalkin S (eds.), Doing Realist Research. Los Angeles; SAGE Publications Ltd. 2018. DOI: 10.4135/9781526451729.n9

[33] Taylor C, Xyrichis A, Leamy MC, Reynolds E, Maben J. Can Schwartz Center Rounds support healthcare staff with emotional challenges at work, and how do they compare with other interventions aimed at providing similar support? A systematic review and scoping reviews. BMJ Open. 2018; 8: e024254. DOI: 10.1136/bmjopen-2018-024254 PMC6196967 30341142

[34] Saul JE, Willis CD, Bitz J, Best A. A time-responsive tool for informing policy making: rapid realist review. Implement Sci. 2013; 8: 103. DOI: 10.1186/1748-5908-8-103 24007206 PMC3844485

[35] Manzano A. The craft of interviewing in realist evaluation. Evaluation. 2016; 22: 342–60. DOI: 10.1177/1356389016638615

[36] Pawson R. Theorizing the Interview. Br J Sociol. 1996; 47: 295–314. DOI: 10.2307/591728

[37] Corless IB, Michel TH, Nicholas M, et al. Educating Health Professions Students About the Issues Involved in Communicating Effectively: A Novel Approach. J Nurs Educ. 2009; 48: 367–73. DOI: 10.3928/01484834-20090615-03 19634261

[38] Shield RR, Tong I, Tomas M, Besdine RW. Teaching communication and compassionate care skills: An innovative curriculum for pre-clerkship medical students. Med Teach. 2011; 33: e408–16. DOI: 10.3109/0142159X.2011.586748 21774636

[39] Stocker C, Cooney A, Thomas P, et al. Schwartz rounds in undergraduate medical education facilitates active reflection and individual identification of learning need. MedEdPublish. 2018; 7. DOI: 10.15694/mep.2018.0000230.1 PMC10712006 38089201

[40] Smith J, Stewart MG, Foggin E, et al. Assessing the benefits and usefulness of Schwartz Centre rounds in second-year medical students using clinical educator-facilitated group work session: not just “a facilitated moan”! BMC Med Educ. 2020; 20: 271. DOI: 10.1186/s12909-020-02199-x 32807145 PMC7433116

[41] Gleeson D, Arwyn-Jones J, Awan M, White I, Halse O. Medical Student Schwartz Rounds: A Powerful Medium for Medical Student Reflective Practice. Adv Med Educ Pract. 2020; 11: 775–80. DOI: 10.2147/AMEP.S273181 33117049 PMC7585519

[42] Abnett H, Tuckwell R, Evans L. Early introduction of the multi-disciplinary team through student Schwartz Rounds: a mixed methodology study. BMC Med Educ. 2022; 22: 523. DOI: 10.1186/s12909-022-03549-7 35786176 PMC9250992

[43] Ho CH. Slowing Medical Education Through Schwartz Center Rounds. Acad Med. 2015; 90: 1290. DOI: 10.1097/ACM.0000000000000865 27002875

[44] Clancy D, Mitchell A, Smart C. A qualitative exploration of the experiences of students attending interprofessional Schwartz Rounds in a University context. J Interprof Care. 2019: 1–10. DOI: 10.1080/13561820.2019.1692797 31821063

[45] Zervos M, Gishen F. Reflecting on a career not yet lived: student Schwartz Rounds. Clin Teach. 2019; 16: 409–11. DOI: 10.1111/tct.13049 31397110

[46] Samad S. A Response to Medical Student Schwartz Rounds: A Powerful Medium for Medical Student Reflective Practice. Adv Med Educ Pract. 2020; 11: 891–2. DOI: 10.2147/AMEP.S291143 33262672 PMC7699997

[47] Smith A. From personal story to group experience: Schwartz rounds give students a safe space to share and learn from the emotional aspects of training. Nurs Stand. 2021; 36: 39–39. DOI: 10.7748/ns.36.6.39.s16

[48] World Health Organization. Working for Health 2022–2030 Action Plan. Geneva: World Health Organization; 2022. Retrieved from https://www.who.int/publications/i/item/9789240063341.

[49] Young P, Moore E, Griffiths G, et al. Help is just a text away: The use of short message service texting to provide an additional means of support for health care students during practice placements. Nurse Educ Today. 2010; 30: 118–23. DOI: 10.1016/j.nedt.2009.06.010 19632011

[50] Hensel JM, Shaw J, Ivers NM, et al. A Web-Based Mental Health Platform for Individuals Seeking Specialized Mental Health Care Services: Multicenter Pragmatic Randomized Controlled Trial. J Med Internet Res. 2019; 21: e10838. DOI: 10.2196/10838 31165710 PMC6684216

