# Supplementary material for: How Does a Group Reflection Intervention (Schwartz Rounds) Work within Healthcare Undergraduate Settings? A Realist Review
Source: Perspect Med Educ. 2023 Dec 19;12(1):550–64. doi: 10.5334/pme.930 (PMC10742148; doi:10.5334/pme.930)
Supplement: Supplementary File 1. — PRISMA diagram, review progression flowchart, annotated (C, M, O) programme theory tables, table of abbreviations, and interview topic guide. [file pme-12-1-930-s1.pdf]

Supplementary file 1

Figure 1 – Flow chart of identification, screening, eligibility and inclusion of literature sources (PRISMA diagram)

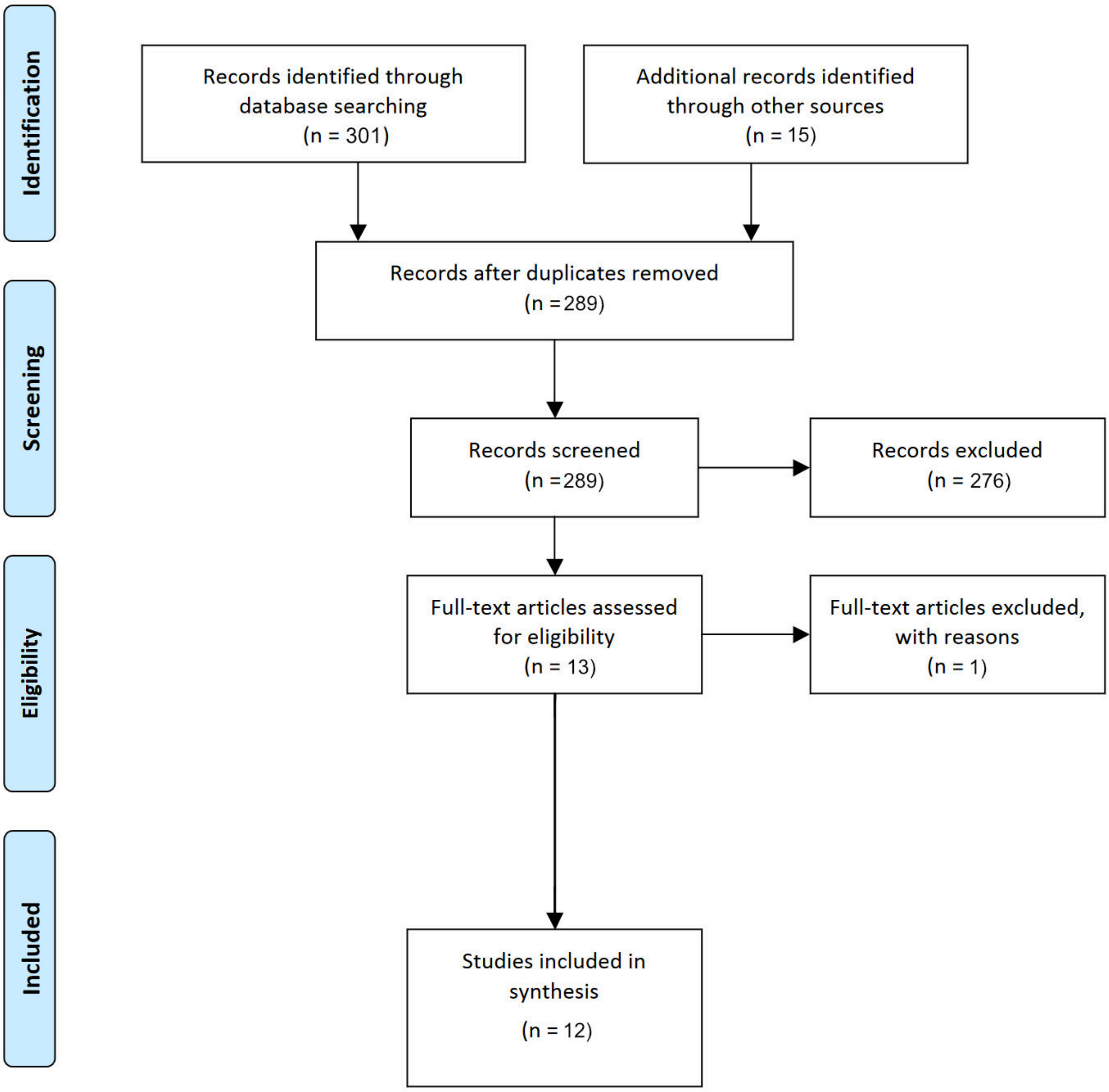

Figure 2 – Review progression

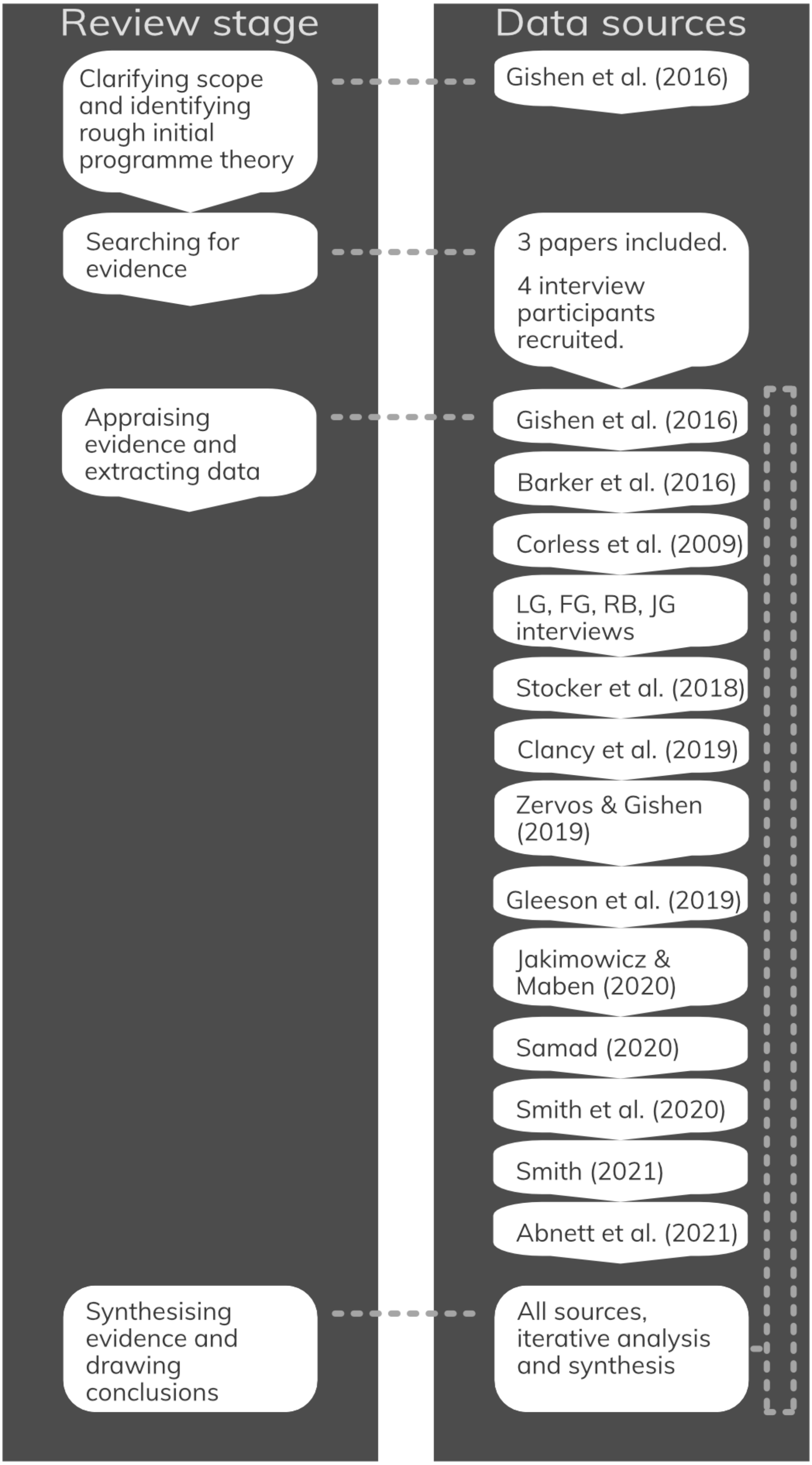

CMO annotation

While reports of realist work frequently label all context-mechanism-outcome configurations (CMOs) with their individual Cs, Ms and Os, we find this can reduce the readability of larger blocks of CMOs, as we present in our paper.

We have formatted our CMOs to try to maintain the ordering within sentences of context, then mechanism, then outcome, wherever possible. However, not all sentences will have a complete CMO. Certain key-words will assist in identifying elements, for example context is typically found as “If…” or “When…” statements.

Please see table 1 and table 2 for annotated CMOs. Please note that decisions for an element being a C, M or O are often arguable and/or dependent on temporality (e.g. when an outcome becomes context in a subsequent CMO).

Table 1

**1 – Rounds provide a reflective space to discuss emotional challenges, thereby enhancing insight and wellbeing**<br>When discussion of difficult topics (e.g. death and disease) in professional care-giving is culturally taboo [C], and when HEIs and students alike focus on teaching and assessment of technical knowledge and skills over social and emotional aspects of care work [C], then a curriculum gap is created around the human side of caring [O].<br>When students are exposed to the challenges of clinical placements during their study [C], then Rounds provide a safe and containing space for reflection on the nature of the work and emotional challenges, to fill the curriculum gap, preparing students for the stresses and demands of professional care work [M], contributing to them being better able to manage their emotions and be resilient, improving student wellbeing [O].<br>When reflection, insight and wellbeing is enhanced [C], then students can successfully maintain empathetic, compassionate practice [O], in part due to a reduction in (or absence of) the symptoms of burnout [M]. This can improve HCP and service-user experiences of care-giving, strengthen communication with both HCPs and service-users, reduce errors attributable to stress, reduce sickness absence and potentially increase retention [O].
**2 – Rounds promote an open and humanised professional culture, enhancing connectedness with colleagues and patients**<br>When professional standards are so high that it is almost impossible to meet those ideals under real-world constraints [C], then experienced HCPs sharing their experiences of vulnerability can recontextualise the meaning of professionalism for students [M], leading to them experiencing less of a distressing disconnect when exposed to practice [O]. When feeling challenged by their work (including early-career imposter syndrome) [C], this allows students to judge themselves less harshly against high professional standards, and realise many professionals experience similar feelings (but mask them well)[O].<br>While Rounds in clinical settings are not explicitly for learning, when Rounds are used in undergraduate settings [C], the educational setting may make some learning goals more explicit [M], such as teaching students about reflection, vulnerability, compassionate care, inter-professional relationships, owning up to mistakes, and showing a more human side [O]. With skilled facilitation which focuses discussion on the impact on the practitioner (not problem solving) [M], such teaching may be achieved without being judgemental or didactic [O].

Open discussion of emotional and social challenges within Rounds [M] can help students realise the benefits of talking things through and being listened to, replacing unhealthy/dysfunctional coping styles (e.g. emotional distancing, alcohol use) with healthy coping behaviours[O]. Students learn to acknowledge and process emotions rather than suppress them, and learn the value of peer-support, promoting connectedness with colleagues[O].<br>Open discussion of emotional and social challenges[M] can also benefit students' learning of advanced communication skills in those areas, blending their personal humanity into professional conversations[O].
**3 – Rounds offer role-modelling of vulnerability which enables greater interpersonal connectedness**<br>When the expression of emotion is culturally suppressed and when panellist stories are from senior clinicians [C], students observe role-modelling of counter-cultural behaviours (openness, vulnerability, courage to have difficult conversations), which offers permission to students to adopt those behaviours [M], including enabling students to share their own stories, within and outside of Rounds [O]. More experienced colleagues are humanised, appearing more approachable and less intimidating, breaking down barriers to communication and bringing HCPs into closer working relationships [O].<br>When students share their own stories, in a Round that focuses on students [C], that disrupts the established hierarchies with teachers and clinicians [M], allowing students to be humanised and not just seen as vessels to be filled with teaching [O].<br>If a panellist is particularly senior or experienced, students' opportunity to hear them talk vulnerably and candidly are rare or non-existent [C]. Students' cultural expectations are therefore subverted [M], enhancing the impact of the story [O]. Similarly, student panellist stories may be less subversive [M]. However, when Rounds includes stories from many levels of experience and seniority, then a more complete range of perspectives [M] paints a fuller picture [O].
**4 – Rounds are impactful when facilitators focus panellists and audience members on emotional and relational elements**<br>If panellists are prepared well, with time dedicated to each panellist by the facilitator to help identify elements of their story most beneficial to a Round, ideally with the other panellists also present [M], then Rounds are more effective, needing less active steering from the facilitator to focus discussion on what the Round is for [O]. However, when panellists are well-prepared[C] then students may feel less confident in following[M] and therefore may decide not

to speak[O]. If panellists are not well-prepared[M], then their story may be too clinically technical, or lack closure[O]. When a student audience desires that closure (reportedly more so than experienced/qualified staff)[C], then time is spent asking questions of the panellists to provide that closure, rather than reflection on the human dimensions of the story[O].
**5 – Rounds offer reflective insights from a wide range of perspectives**<br>If panellist stories are drawn from multiple disciplines [C], then students are exposed to a range of perspectives on a case or theme [M], giving students greater insight on HCP and service -user experiences, that goes beyond students' individual experiences [O].<br>Group reflection promotes compassion and empathy among students [M], with the examination of past experiences providing a guide for future events [O].<br>When teachers or clinicians are present [C], discussion in Rounds generates insights for them, in addition to students [O].

Table 2

**6 – Rounds allow reflection to be more engaging for students when they are non-mandatory**<br>When Rounds are included as part of the curriculum [C], mandatory attendance should not be considered long-term, as the style of reflection will not suit all students and, through losing an important core feature of Rounds (voluntary attendance) [M], the nature and tone of Rounds may be detrimentally changed [O]. However, when Rounds are complex and countercultural [C], initially mandating students attend a small number of Rounds could help students understand their purpose and value [M], increasing attendance from students who will find Rounds valuable [O].<br>If Rounds are non-mandatory [C], then students can engage more enthusiastically with reflection, as compared to mandatory written reflections that are typical of HCP training [M], leading to different insights and making the reflection more effective [O]. However, when Rounds are a non-mandatory, non-assessed activity [C], students may not immediately understand their value, requiring some time to adapt to and accept [O].<br>When opportunities for reflection are limited within a curriculum, if Rounds are scheduled as part of curricular time [C], then a larger scale, confidential forum for reflection is available to students [M], creating more opportunities for students to participate in reflection [O].
**7 – Perceptions of safety within a Round varies based on multiple factors**<br>When the audience size of in-person Rounds is not so large [C] as to be intimidating for students to share personal stories (35 to 50 people), then audience participation is likely to be higher [M], increasing disclosure from students and therefore enabling reflective insights [O]. At this audience size [C], everyone present can see and sense each other comfortably [M], increasing the sense of safety and allowing for facilitators to be more effective, both during and after the Round [O]. Without this limit on audience size [C], discussions may happen instead in post-round ad hoc conversations, within smaller peer groups, without the safety of professional facilitation [O].<br>If the audience is multidisciplinary [C], then students are exposed to a broader range of perspectives and recognise experiences in common [M], promoting connectedness and understanding of interprofessional working [O]. If the audience is unidisciplinary [C], then having a range of panellists from different professions [M] can still provide a valuable range of perspectives [O]. If the audience is unidisciplinary [C], then they may feel freer or more confident to speak up and contribute their own story to the Round [O].

When Rounds are run solely for a student audience [C], then the reduced perception of hierarchies found in clinical practice allows students to have courage to make their voices heard[M], creating a safer space to share thoughts with confidence, compared to clinical Rounds where students can feel less "safe" to speak[O].

Student confidence to share[O] depends on a sense of safety and containment[M], which can be affected by clinical experience, a younger age, the pace of conversation, willingness to tolerate emotional discomfort, and fear of judgement from peers and teaching staff[C].

8 – Adapting timing and themes to students' changing needs may improve engagement

Over a course of study, students' interests, short term priorities (such as assessments) and level of clinical experience varies [C]. When this context is taken into account, Rounds themes and timing within the curriculum can be tailored to students' interests and needs [M], improving engagement and attendance [O].

9 – Resonance with Rounds' stories is affected by clinical experience levels

When students lack clinical experience [C], then they may not have acquired the contextualised knowledge to be able to respond as meaningfully to panellist and audience stories [M], reducing the value of that reflection [O]. As students acquire clinical experience, that experience increases the resonance of Rounds stories[M], as well as increasing students' need for the benefits of Rounds [O].

However, when Rounds are trying to change the culture of conversations, and when students already feel pressured by judgement and scrutiny [C], then introducing Rounds as early as possible (e.g. inclusion of first-years) allows more time [M] for cultural change to happen [O]. When lack of clinical experience may be a limiting factor, then using a more generic theme or one related to student experience can still enable students to engage in relationally focused conversations, and consequently benefit from them.

When students qualify and gain more clinical experience of emotional and relational challenges, they can apply insight from Rounds attended as an undergraduate, revealing new understanding of stories upon applying the reflective learning in practice.

10 – Recorded or online Rounds can increase reach but presents risks for psychological safety

Through video recording and sharing Rounds online [M], further benefits can be realised such as distance learning and reincorporation of material into the

taught curriculum, expanding access to Rounds' insights [O]. However, the act of recording may inhibit the confidentiality and perceived safety of the Round, reducing attendees' willingness to share their stories, or alter the openness and honesty with which they do so[O].

When a Round is not experienced in-person[C], then auditory and visual cues (body language, facial expressions) from others cannot be easily experienced[M], inhibiting emotional connection and understanding[O]. If the Round experience is online[C], that lack of direct contact[M] can reduce awareness and opportunity for facilitators to follow up with anyone who may be especially distressed by the Round discussion, meaning extra "safety net" processes may be necessary[O].

Table 3 – Abbreviations

Abbreviation	Expansion
CMO	Context-mechanism-outcome (configuration)
HCP	Healthcare professional
HEI	Higher education institution
NHS	National Health Service
POCF	Point of Care Foundation

Topic guide

Introduction

- Introduce self
- Reminders about confidentiality, consent and withdrawal. Confirm understanding with participant.
- Recap research area and intent.
- Brief explanation of realist interviewing: what it is for, why is it different to interviews the participant might have been involved in previously, the teacher-learner cycle to test theory.
  - Explain much of the interview will focus on why/how type questions, that this is not meant to challenge the participant but is necessary to dig deeper into the subject.
- Any questions or concerns?

Outline

- Why run Rounds for students? What are Rounds "for" in this setting?
  - Different to clinical settings? How? Why?

    - Role and responsibility of the university
    - Is this the same or different across the range of healthcare courses? Any group that responds in a different way?
    - Effects at different levels? Personal, group, organisation? What? How are they produced?
    - Are Rounds merely beneficial, or necessary? (curriculum gap) To what extent? Why?
    - Resilience
    - Compassion / compassionate behaviours
        - Other HCPs
        - Patients, families, carers
- Can you tell me a bit about your sense of how students respond to Rounds?
    - Reflection
    - Sharing stories
    - In the Rounds and after the Round
    - One off versus cumulative effects? What are they? How do Rounds provide that effect? Why is that important for students?
    - Any noticeable differences in any particular group of students? What? Why?
    - What about students who aren't attending Rounds? Who? Why? Do you consider that problematic? Why?
- Can you tell me about a Round for students that was particularly powerful? What happened?
    - What makes this Round different, or stand out? Why?
        - Theme
        - Panel

        - Audience
        - Facilitation
- Do you remember a student Round that didn't feel as impactful as you were expecting?
    - What makes that Round different? Why?
        - Theme
        - Panel
        - Audience
        - Facilitation
- Have student Rounds evolved at all over time?
    - How? Why? (or why not?)
    - Rounds savviness, developing culture of Rounds? What? How?
    - Adaptations? What? Why?
        - Theme
        - Panel
        - Audience
        - Timing

Conclusion

- Is there anything we haven't spoken about that you had expected to discuss? Or something you'd like to mention that you haven't been asked?
- Possibility of follow up questions/discussion later.
- How to stay informed about the study progress.
- Thanks and appreciation.
